# Mortality impact of an increased blood glucose cut-off level for hypoglycaemia treatment in severely sick children aged 5 to 12 years in Malawi – an exploratory randomised controlled study

**DOI:** 10.1186/s12887-026-06660-6

**Published:** 2026-03-03

**Authors:** Fatsani Ngwalangwa, Margaret Nyaika, Tim Baker, Queen Dube, Josephine Langton, Helena Hildenwall

**Affiliations:** 1 Department of Epidemiology and Biostatistics, Kamuzu University of Health Sciences, Blantyre, Malawi; 2 Mercy James Paediatric Surgical and Intensive Care unit, Queen Elizabeth Central Hospital, Blantyre, Malawi; 3 Department of Anaesthesia and Intensive Care, Queen Elizabeth Central Hospital, Blantyre, Malawi; 4 Department of Emergency Medicine, Muhimbili University of Health and Allied Sciences, Dar es Salaam, Tanzania; 5 Department of Global Public Health, Karolinska Institutet, Stockholm, Sweden; 6 Department of Paediatrics, Queen Elizabeth Central hospital, Blantyre, Malawi; 7 Department of Paediatrics and Child Health, Kamuzu University of Health Sciences, Blantyre, Malawi; 8 Center for Health and Sustainability, Department of Women’s and Children’s Health, Uppsala University, Uppsala, Sweden; 9 Department of Clinical Science, Intervention and Technology, Karolinska Institutet, Huddinge, Sweden

**Keywords:** Hypoglycaemia, Low glycaemia, Dextrose treatment, Severely sick children, critical illness

## Abstract

**Background:**

Low blood glucose concentration is a well-recognized risk factor for mortality in children admitted to hospitals in low-income settings. Current WHO guidelines define hypoglycaemia as a blood glucose level below 2.5 mmol/L; however, this threshold has been questioned. In a trial conducted in Malawian hospitals, increasing the treatment threshold to 5.0 mmol/L did not reduce in-hospital mortality among children aged 1 month to 5 years. The physiological response to low glucose and dextrose treatment in older children may differ yet remains understudied. This exploratory analysis was conducted to assess potential effects of dextrose treatment in severely ill children aged 5–12 years with blood glucose concentrations between 2.5 and 5.0 mmol/L.

**Methods:**

The study is an exploratory extension of a pragmatic randomised controlled trial where all eligible children were randomised to receive either a 5 ml/kg intravenous bolus followed by maintenance infusion of 10% dextrose (intervention group) or observation and standard care (control group). The primary outcome was in-hospital mortality; the secondary outcome was 24-hour mortality.

**Results:**

Seventy-two children were enrolled with 36 allocated to each group. In-hospital mortality was 8% (3/36) in the intervention group and 19% (7/36) in the control group with an odds ratio (OR) for mortality of 0.8 (95% CI 0.46–2.25). The mortality after 24 h was 3% (1/36) in the intervention group and 8% (3/36) in the control group, with an OR of 0.4 (95% CI. 0.06–2.90). Adverse events occurred in 19% (7/36) in the intervention group and 39% (14/36) in the control group.

**Conclusion:**

This exploratory analysis suggests a potential reduction in mortality when intravenous dextrose treatment is administered to severely ill children aged 5–12 years who present to hospital with a low blood glucose. However, as the parent trial was powered for children less than 5 years the sample size for this older age group was insufficient to draw definitive conclusions. Larger, age-specific studies are warranted to determine the potential benefit of dextrose treatment in this population.

**Trial Registration:**

The study is registered with ClinicalTrials.gov, NCT02989675 on 7th December 2016.

**Supplementary Information:**

The online version contains supplementary material available at 10.1186/s12887-026-06660-6.

## Background

Hypoglycaemia is recognised as a medical emergency which may occur in severely ill children presenting to hospitals and especially in low income countries [1]. It is associated with an increased risk of in-hospital mortality with a case fatality in admitted febrile children of up to 42% compared to 3% among those with normal blood glucose [2]. Severely sick children are at a risk of hypoglycaemia, but the reasons are insufficiently understood and could probably be due to poor feeding, increased glucose demand and/or metabolic disturbances that commonly occur in severe illness [3–5]. The World Health Organisation (WHO) defines hypoglycaemia as a blood glucose level of less than 2.5mmol/l, or 3.0mmol/l in severely malnourished children, and these cut-offs are used as a threshold for the decision to provide treatment [6]. However, there are several controversies and debates with this definition [7] with reports of an increased mortality also among children who present with a blood glucose level that is higher than the WHO cut-off of 2.5 mmol/l [2, 8]. Several alternative cut-offs have been used to define low glycaemia, and a blood glucose level ranging from 2.2mmol/l up to 5.0 mmol/l has been shown to be associated with increased mortality risk [8–11]. Higher mortality among children admitted with low glycaemia could be due to progression to unnoticed hypoglycaemia when blood sugar monitoring is not done [12].

A recent study that was providing dextrose treatment to severely ill children aged 1 month to 5 years who presented with a blood glucose concentration of 2.5–5.0 mmol/l did not demonstrate any impact on mortality [13]. However, older children may have a different glucose metabolism compared to younger children and could possibly adapt better to glucose treatment [14, 15]. Hence, this exploratory study was conducted to assess the impact of dextrose treatment on severely sick children admitted to hospital with a blood glucose level between 2.5 and 5.0 mmol/l in children aged 5–12 years. Children aged 5–12 years are an understudied group and data on their potential response on hypoglycaemia and its treatment has not been reported. We therefore conducted this study to get data that may inform future studies on dextrose treatment and blood glucose cut-off.

## Methods

### Study design

This study was conducted as an explorative extension of the SugarFACT trial, which primarily aimed to assess the impact of hypoglycaemia treatment on in-hospital mortality among severely ill children aged 1 month to 5 years with blood glucose concentrations between 2.5 and 5.0 mmol/L—above the WHO threshold for treatment. The present analysis was carried out in parallel with the main trial and involved children aged 5 to 12 years, who were enrolled to provide preliminary insights into whether a revised treatment threshold might benefit older children. As the trial was powered only for the younger age group, the inclusion of older children was intended to generate hypotheses for future study. The study enrolled children from December 2016 to January 2019. Results from the main trial are presented elsewhere [13].

### Study setting

The study was conducted at two referral hospitals in Malawi: Queen Elizabeth Central Hospital (QECH) in Blantyre and Zomba Central Hospital (ZCH). QECH is the largest tertiary referral hospital located in the southern region of Malawi, and it is also a teaching hospital admitting 23,000 children annually. ZCH is also a tertiary hospital located in the eastern region of Malawi with an estimated 2500 paediatric admissions annually. As tertiary referral facilities both hospitals serve both the rural and the urban population. The Malawi government rolled out the Emergency Triage, Assessment and Treatment guidelines (ETAT) in all the hospitals in Malawi in 2001 [16]. ETAT is a tool to assist in the identification of severely ill children for immediate treatment and stabilisation upon arrival to hospital. QECH has a 12-bed paediatric Intensive Care Unit (ICU) reserved for the most critically ill children. It also has a High Dependency Unit (HDU) for those with severe but stable conditions. When the ICU capacity is exceeded, some critically ill children are temporarily admitted to the HDU. Zomba Central Hospital has an ICU which serves both adult and paediatric patients. In addition, there is a paediatric HDU with eight beds, serving critically ill children.

### Participants

**Fig. 1:**
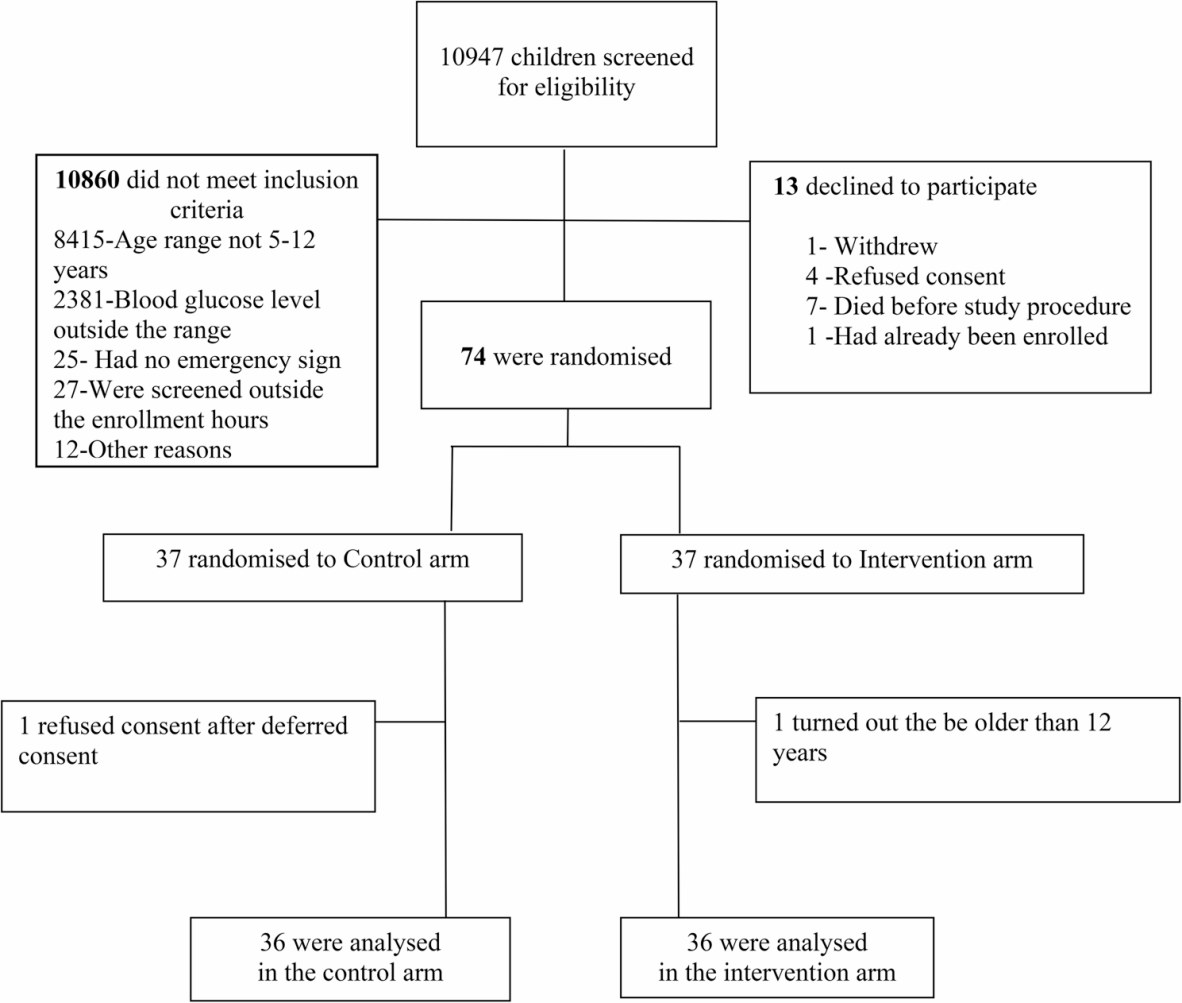
Trial Profile

Children aged between 5 and 12 years were eligible for inclusion in the study if they presented to one of the study hospitals with any WHO defined emergency sign as follows: (1) Obstructed or absent breathing (2) Central cyanosis (3) Severe respiratory distress (4) Coma (5) Convulsion (6) Severe dehydration and (7) Shock, defined by the presence of all the following: cold extremities, capillary refill of > 3 s, and a weak and fast pulse. Clinical concern was also added as a danger sign, and this was defined as a child without any of the above stated WHO emergency signs, but the admitting clinician considered the child to be severely ill. The inclusion criteria also included an admission blood glucose level of 2.5–5.0 mmol/l. Children with a known diagnosis of diabetes mellitus, whose caretaker refused consent or who had already been enrolled in the study in a previous admission were excluded from study enrolment (Fig. 1).

### Procedures

Participants were recruited from the QECH emergency department and from ZCH outpatient and admitting unit where all sick children arriving at the facilities are first seen before admission. The recruitment time was 07:00–21:00 during the weekdays and 08:00–16:30 on weekends. The study staff were based in the admission area to screen and enrol eligible participants. The routine staff at the emergency department and admission units in the two hospitals were also screening participants outside the enrolment hours to provide information on the numbers of participants screened after recruitment hours. The study staff included clinical officers and nurses.

All severely ill children in the two hospitals had a capillary blood glucose concentration assessed on arrival to the hospital using a HemoCue Glucose 201 RT point-of-care glucometers. The HemoCue glucose 201 RT has been used in other settings and showed to have reliable performance [17]. Weekly quality control checks of the HemoCue glucometers were conducted using GlucoTrol-NG control fluids (Eurotrol B.V, Keplerlaan 20,6716 BS Ede, The Netherlands).

### Randomisation

Participants were 1:1 randomly assigned to intervention group and control group. A statistician produced a computer-generated stratification list by study site using variable block sizes of six and eight a priori to the study start. When an eligible participant was identified, study personnel revealed the group allocation by opening a sealed opaque envelope in strict sequential order and thereby assigning the study identification number for the participant. Study numbers appeared on all case report forms and source documents. However, group allocation was concealed during data analysis. Children were randomised to receive either the intervention, i.e., a bolus of 5 ml/kg 10% dextrose followed by a maintenance infusion of 10% dextrose, or control i.e., no dextrose treatment as per standard of care for any child presenting with blood glucose of > 2.5mmol/l. The randomisation was done separately from the main study.

For all enrolled in the intervention group, a capillary blood glucose was re-checked 30-minutes after the first bolus of 5 ml/kg 10% dextrose and a repeat bolus of dextrose was given if the blood glucose concentration after the first bolus remained at less than 5.0 mmol/l. Repeat checks were done every 30 min as per standard procedures for all patients who received dextrose treatment due to low blood glucose. Boluses were given until measurement levels were 5.0 mmol/l or above. Children in the control group were given the standard care as per WHO recommendations with no boluses of dextrose and decisions on any IV or NG fluids as per the clinician’s assessment.

All severely malnourished children were treated with maintenance glucose-containing fluids via nasogastric tube as per standard care. All other treatments were conducted as per the hospital’s routine standard of care, meaning children in the control group only had their blood glucose checked if a clinician specifically asked for it, which could occur in case of symptoms suggesting hypoglycaemia. No repeat blood glucose checks in the control group were scheduled for study purposes.

A follow-up was conducted by the study team 24 h after enrolment and daily until discharge. Data collected at 24 h included any repeat blood glucose measurements in the wards, the total dextrose received intravenously and by nasogastric tube, any food or sweet drinks received and the occurrence of any Serious Adverse Events (SAEs). The daily follow-ups collected information on outcomes and discharge diagnoses as well as SAEs.

All study staff were trained in ETAT and Good Clinical Practice and regular refresher training of study procedures were conducted. The HemoCue glucometer, cuvettes, dextrose and intravenous fluids were provided by the study.

### Outcomes

In-hospital mortality was the primary outcome whilst 24-hour in-hospital mortality was the secondary outcome.

### Serious adverse events (SAEs)

Adverse events were monitored daily by study staff and reported to the study coordinator as soon as noticed. Since the study population was severely sick and several adverse events were expected, only serious adverse events were reported. The following were considered as serious adverse events; reduced coma score (defined as a Blantyre coma score of less than 5), convulsion, hypoglycaemia (blood glucose level of < 2.5mmol/l), hyperglycaemia (blood glucose of > 10mmol/l) or death. SAEs were reported by the study team to an independent clinical monitor for assessment of a potential relationship with the study intervention within 48 h. The SAEs were sent to the clinical monitors without details of the treatment group to minimise bias during the assessment. The Data Safety and Monitoring Board (DSMB) and the ethical review board also received the SAE but after the assessment by the clinical monitors.

### Sample size

The enrolment of children aged 5 to 12 years was conducted in parallel to the main SugarFACT trial. As the primary sample size calculation was designed to power the trial for children aged 1 month to 5 years, no formal power calculation was performed for this older age group. Inclusion of children aged 5–12 years was intended for exploratory purposes only, to provide preliminary insight into the potential impact of dextrose treatment in this age group.

### Data management and statistical analysis

Electronic Clinical Record Forms (eCRFs) were developed using Open Data Kit (https://opendatakit.org) on Android tablets. Data were uploaded daily to a database overseen by a data manager and data queries were raised and resolved with regular meetings between the investigators and data manager. Data analysis was conducted using Stata version 14. Baseline characteristics are presented with median and proportions where appropriate. Chi Square test and t-test were also used to determine if there were any significant differences in baseline characteristics between the two groups. Due to the small sample size, firth logistic regression was conducted to determine the association between the intervention and in-hospital mortality.

## Results

From December 5 2016, through January 22 2019, 10,947 children presented to the Paediatric Emergency Departments at QECH and ZCH of these, 2532 (23%) were within the age range of 5–12 years of which 74 (0.7%) children were aged 5 years to 12 years and had either a WHO defined emergency sign or a clinical concern that the child’s condition was an emergency combined with a blood glucose of 2.5–5.0 mmol/l. Of these, two children were further excluded from the analysis because one refused full consent after the deferred consent had been provided and the other one was noted to be older than 12 years after mistakenly randomizing them. The remaining 72 patients were randomly assigned to Control (*N* = 36) or Intervention (*N* = 36). (Fig. 1)

### Background characteristics of study participants

A total of 33 (46%) of the eligible children were recruited from QECH and 39 (54%) were recruited at ZCH. The mean age was 6.5 years (Range 5.6–7.8) and 58% were male. A total of 11 (16%) children were assessed as being malnourished. The median blood glucose concentration on arrival to hospital was 4.5 mmol/L (IQR 3.8–4.7mmol/l) in the control group and 4.1 mmol/l (IQR 3.6–4.3 mmol/l) in the intervention group. Severe respiratory distress was the most common presenting emergency sign with 7 (19%) in the control group and 9 (25%) in the intervention group. None of the participants presented with obstructed or absent breathing.

### Effect of dextrose treatment on mortality

A total of 10 out of the 72 participants died representing a mortality of 14% in the study group. For the primary outcome of all-cause in-hospital mortality, 7 (19%) of the 36 children in the control group and 3 (8%) of 36 children in the intervention group died (mortality difference 11%). The odds ratio (OR) for death among children in the intervention group was 0.8 [95% Confidence Interval (CI) 0.5–2.3] compared to that for children in the control group but this was not statistically significant. At 24 h after enrolment, 3 (8%) of 36 children in the control group and 1 (3%) of 36 children in the intervention group had died in the first 24 h of arrival to the health facility (mortality difference of 5%, OR 0.4 (95% CI 0.06–2.90)) (Tables 1, 2 and 3). The distribution of repeat glucose tests and oral feeds during the admission were similar between control and intervention group but differed in total grams/kg of dextrose received through IV or NG route (Supplementary Table 1).

**Table 1:** Baseline Characteristics of the study participants

Variable	Control group (*N* = 36) *n* (%)	Intervention group (*N* = 36) *n*(%)	Total (*N* = 72) *n*(%)	*p*-value
Recruitment site				
QECH	16 (44)	17 (47)	33 (46)	0.81
ZCH	20 (56)	19 (53)	39 (54)	
Malnutrition	6 (17)	5 (14)	11(16)	0.74
Age, median years *(Interquartile Range)	7.1(5.8–8.7)	6 (5.3–7.0)	6.5 (5.6–7.8)	0.63
Sex				
Female	14 (39)	16 (44)	30 (42)	0.63
Male	22 (61)	20 (56)	42 (58)	
Emergency Signs				
Cyanosis	0 (0)	3 (8)	3 (4)	0.08
Severe respiratory distress	7 (19)	9 (25)	16 (22)	0.57
Shock	0 (0)	2 (6)	2 (3)	0.15
Coma	4 (11)	9 (25)	13 (18)	0.13
Convulsions	9 (25)	5 (14)	14 (19)	0.23
Dehydration	1 (3)	2 (6)	3 (4)	0.56
Clinical Concern	18 (50)	14 (39)	32 (44)	0.34
HIV test				
Negative	14 (39)	15 (42)	29 (40)	0.94
Positive	6 (17)	5 (14)	11(15)	
Unknown	16 (44)	16 (44)	32 (44)	
Positive rapid diagnostic test for malaria	18 (50)	14 (40)	32 (45)	0.40
Blood glucose at arrival, mmol/L* (median) (Interquartile range)	4.5 (3.8–4.7)	4.1(3.6–4.3)	4.3(3.7–4.6)	0.03

*p value was obtained using t-test, the rest were obtained using Chi-square test

**Table 2:** Effects of dextrose treatment on in-hospital mortality and 24-hour mortality

	Control Group	Intervention Group	Odds ratio	*P*-value*
	*(N*=36)	(*N* = 36)	(95%CI)	
In hospital deaths	7 (19%)	3 (8%)	0.8 (0.5–2.3)	0.20
Death at 24 h after enrolment	3 (8%)	1 (3%)	0.4 (0.1–2.9)	0.37

*p values and Odds ratio were obtained using firth logistic regression

**Table 3:** Serious Adverse Events

	Control group (*N* = 36)	Intervention group (*N* = 36)	Total (*N* = 72)	*p*-value*
Hypoglycaemia	0 (0%)	1 (2.8%)	1 (1.4%)	0.47
Convulsion	3 (8.3%)	3 (8.3%)	6 (8.3%)	1.00
Reduced consciousness	3 (8.3%)	1 (2.8%)	4 (5.6%)	0.31
Death	7 (19.4%)	3 (8.3%)	10 (13.9%)	0.78

*Chi square *P* value

### Serious adverse events

A total of 21 serious adverse events were reported, with 7/36 (19%) occurring in the intervention group and 14/36 (39%) in the control group. One episode of hypoglycaemia (< 2.5mmol/l) occurred in the control group and none in the intervention group. There were four (19%) serious adverse events of reduced consciousness whereof three (8.3%) occurred in the control group and one (2.8%) in the intervention group. There were no reported cases of hyperglycaemia in the study.

## Discussion

This exploratory analysis suggests a potential benefit of dextrose treatment among children aged 5–12 years who were admitted with a severe illness and had a blood glucose level of 2.5mmol/L to 5.0mmol/L compared to children who did not receive dextrose treatment. However, the benefit is only observed in the absolute difference between proportions and cannot be statistically confirmed due to the small sample. This warrants a need for a larger confirmatory trial that may help change future practice in the blood glucose cut-off for administering dextrose treatment. Blood glucose level above the WHO cut-off levels has been shown to contribute to poor outcomes in severely ill children [1, 2]. Timely interventions, especially in low resource settings like Malawi may reduce mortality and other poor outcomes.

The findings stand in contrast to the results of the SugarFACT trial [13], which found no mortality benefit from raising the treatment threshold for hypoglycaemia in severely ill children aged 1 month to 5 years. A difference between younger and older children is not unexpected, given what is currently known regarding glucose metabolism in different age groups. It is generally assumed that young children are more susceptible to hypoglycaemia as young children have a higher glucose requirement compared to older children and use up their glucose faster than older children [14, 15]. Studies indicate that the peak brain glucose requirement occurs at around 4 years of age, with the brain consuming approximately 66% of the body’s resting metabolic rate, compared to only 43% at age 10 [16]. In healthy children, a 5% maintenance dextrose infusion covered a larger proportion of the overall glucose requirement in older children up to 18 years compared to younger children up to one year [17]. Young children demonstrate impaired counterregulatory hormone responses to hypoglycaemia compared to older children [14, 15]. In addition, younger children have limited storage of glucose compared to older children and could thus be assumed to benefit more from getting glucose treatment [14]. However, the results of the present study indicate, though cannot confirm, the opposite relationship, suggesting that older children may derive greater benefit from a changed hypoglycaemia treatment cutoff. We hypothesize that a developed counterregulatory hormone system in older children enables better coordination of glucose homeostasis following IV glucose administration [15–17].

For the adverse events, children in the control group had a higher occurrence of hypoglycaemia and reduced consciousness compared to those in the intervention group. This further suggests some benefits of administering dextrose to also prevent episodes of hypoglycaemia and its consequences in the ward. The importance of glucose monitoring in those with low glycemia and providing maintenance fluids to any child with insufficient intake to avoid hypoglycaemic events during admission should also be emphasized [18].

This study has several limitations that should be acknowledged. First, the sample size was small, reflecting the exploratory nature of the analysis and the fact that data collection for this age group was conducted in parallel to the main trial, which was powered for younger children. As a result, the study lacks the statistical power to draw definitive conclusions, and the findings should be interpreted with caution. Second, randomisation did not result in fully comparable groups: the median admission blood glucose concentration was higher in the control group than in the intervention group, which may have introduced bias and affected the observed outcomes. Reasons for this difference in blood glucose are unknown, as proper randomization procedure were conducted using sealed envelopes that were not opened until a child had been selected for inclusion in the study. Also, the randomisation of blood glucose concentration levels was successful in the main study, in which a larger number of children were included, whereby the same staff and randomisation procedures were used. The small sample size in this study could have possibly contributed to the observed differences. Use of clinical concern as an emergency sign in addition to the WHO established emergency signs might have diluted the severity of the illness in the study population. However, the group was added as the investigators agreed that there may be times when a clinician has a clear feeling that a child is severely sick despite not fulfilling any established criteria for severe illness. For that reason, we decided to also allow for clinical concern to be part of inclusion criteria.

Despite the difference in admission blood glucose concentration, other variables were well balanced between groups, and this is a strength of this study. Efforts were made to capture all types of energy provided to the study group whereby information was collected on oral nutrition intake, total bolus, and maintenance of glucose received whether in nasogastric tubes or intravenously.

## Conclusions

In this exploratory analysis of children aged 5 to 12 years the provision of dextrose treatment using a blood glucose cutoff level of 5.0 mmol/L suggested a possible mortality reduction and fewer adverse events compared to standard care. While these findings suggest a potential clinical benefit, the small sample size limits the strength of the conclusions. These preliminary results highlight a potential age-related difference in response to dextrose treatment in severe illness that warrants further investigation to understand the impact of dextrose treatment in older children with low glycaemia in adequately powered trials.

## Supplementary Information

Supplementary Material 1.

## Data Availability

The datasets used and analysed during the current study are available from the corresponding author on reasonable request.

## Abbreviations

COMREC: College of medicine research ethics committee
DSMB: Data safety and monitoring board
ECRF: Electronic case reporting forms
ETAT: Emergency triage, assessment and treatment guidelines
QECH: Queen Elizabeth Central hospital
SAE: Serious adverse event
WHO: World health organisation
ZCH: Zomba Central hospital
