# Supplementary material for: Mortality impact of an increased blood glucose cut-off level for hypoglycaemia treatment in severely sick children aged 5 to 12 years in Malawi – an exploratory randomised controlled study
Source: BMC Pediatr. 2026 Mar 3;26:276. doi: 10.1186/s12887-026-06660-6 (PMC13064174; doi:10.1186/s12887-026-06660-6)
Supplement: Supplementary file 1 — Supplementary Material 1. [file 12887_2026_6660_MOESM1_ESM.docx]

**Supplementary table 1: Dextrose treatment and glucose intervention within 24 Hours Post-Admission**

|  | **Control Group** | **Intervention group** | **p-value** |
| --- | --- | --- | --- |
| Blood sugar rechecked in the ward in 24hrs, n (%) | 8 (22.2) | 8 (22.2) | 1.00 |
| Total grams/kg dextrose received* through IV or NG route in first 24hrs (mean+/-SD) | 0.4 (1.1) | 7.5 (12.7) | <0.05 |
| Received one or more bolus of dextrose in the ward in 24hrs, n (%) | 3 (8.6) | 2 (5.6) | 0.68 |
| Received NG feeds/dextrose containing in the ward, n (%) | 2 (5.6 | 2 (5.6 | 1.00 |
| Oral feed received in the first 24   hours(including sweet drinks, milk, food), n (%) | 26 (72.2) | 29 (80.6) | 0.41 |

*Total grams/kg dextrose excludes the first bolus of dextrose given
